# Next-generation sequencing reveals how RNA catalysts evolve from random space

**DOI:** 10.1093/nar/gkt949

**Published:** 2013-10-23

**Authors:** Sandeep Ameta, Marie-Luise Winz, Christopher Previti, Andres Jäschke

**Affiliations:** ^1^ Institute of Pharmacy and Molecular Biotechnology (IPMB), Heidelberg University, 69120-Heidelberg, Germany and ^2^High Throughput Sequencing Core Facility, German Cancer Research Center (DKFZ), 69120-Heidelberg, Germany

## Abstract

Catalytic RNAs are attractive objects for studying molecular evolution. To understand how RNA libraries can evolve from randomness toward highly active catalysts, we analyze the original samples that led to the discovery of Diels–Alderase ribozymes by next-generation sequencing. Known structure-activity relationships are used to correlate abundance with catalytic performance. We find that efficient catalysts arose not just from selection for reactivity among the members of the starting library, but from improvement of less potent precursors by mutations. We observe changes in the ribozyme population in response to increasing selection pressure. Surprisingly, even after many rounds of enrichment, the libraries are highly diverse, suggesting that potential catalysts are more abundant in random space than generally thought. To highlight the use of next-generation sequencing as a tool for *in vitro* selections, we also apply this technique to a recent, less characterized ribozyme selection. Making use of the correlation between sequence evolution and catalytic activity, we predict mutations that improve ribozyme activity and validate them biochemically. Our study reveals principles underlying ribozyme *in vitro* selections and provides guidelines to render future selections more efficient, as well as to predict the conservation of key structural elements, allowing the rational improvement of catalysts.

## INTRODUCTION

RNA, although a simple molecule, possesses a high catalytic potential. Initially found to occur naturally (1,2), ribozymes catalyzing a wide range of chemical transformations (3–7) have been isolated using combinatorial *in vitro* selections (8,9). In these experiments, a population of different RNAs (typically ∼10^14^ sequences) is challenged for a specific task, and the selection process is designed such that few active sequences are retained and then enzymatically amplified. To observe a significant enrichment of active sequences over background, 8–15 iterative rounds are usually conducted, and mutational errors in the amplification steps are assumed to make this a true evolutionary process in which species evolve that were not contained in the starting population (10). Evidence for this claim is, however, scarce, mainly because no methods existed for analyzing mixtures of this enormous complexity. Although there are elegant demonstrations of how one functional RNA sequence can be evolved to changed ion specificity (11) or to carry out a different function by a series of mutations (12), the pathways evolution has actually taken in selection experiments are largely unknown. Similarly, there is no certainty about how RNA populations react to changes in selection pressure, and how precisely the composition and diversity vary over the selection cycles.

Current next-generation sequencing (NGS) technology allows millions of relatively long nucleic acids to be read at once (13,14). Recently, NGS was used for analyzing *in vitro* selections of protein binding or inhibiting nucleic acid aptamers and functional proteins (15–19). Although NGS has been used to construct the fitness landscape of a ligase ribozyme (20), it has so far not been used to study ribozyme evolution from random sequence and structure space.

**Figure 1.**
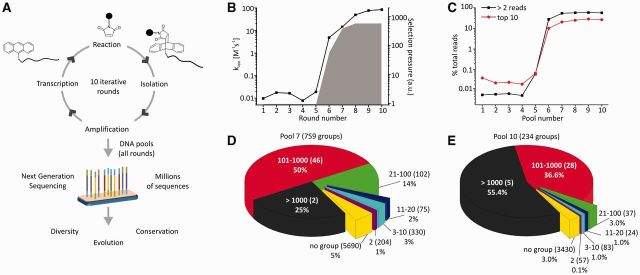
*In vitro* selection and pool diversity. (**A**) *In vitro* selection of DAse ribozymes and their analysis by NGS. (**B**) DAse ribozyme selection profile, showing apparent rate constant *k*_app_ and relative selection pressure over the rounds (see Supplementary Text S1). (**C**) Numeric sequence diversity of different DNA pools, measured by the fraction of total reads covered by sequences having >2 reads and by the 10 top ranking sequences of each pool. (**D** and **E**) Structural diversity of pools 7 and 10, respectively, measured by fraction of total reads covered by sequence groups with different numbers of members (number of groups in parentheses).

More than a decade ago, Diels–Alderase (DAse) ribozymes were selected for catalyzing the eponymous cycloaddition (Figure 1A and B) (6). Active sequences isolated after 10 iterative rounds were rationally minimized to yield a 49mer DAse ribozyme, which has been characterized thoroughly (21–25), providing a good understanding of structure–function relationships. Lately, we selected a different ribozyme, which selectively and site-specifically reacts with a mechanistic inhibitor of serine proteases (3). The mechanistic inhibitor reactive ribozymes (MIRzymes) were selected in 13 rounds of selection (Supplementary Figure S1). The covalent adduct formed between the inhibitor and MIRzyme shows high similarity with that formed between inhibitor and serine proteases. 

Here, to understand the evolutionary process of ribozymes during *in vitro* selections, the DNA pools from the individual rounds of both original selections (3,6) were subjected to NGS, followed by sequence- and structure-based analyses. These analyses revealed the pathways that the ribozymes followed during the *in vitro* selection process and allowed us to study as well as predict nucleotide conservation in key structural elements.

## MATERIALS AND METHODS

All enzymes and reagents were from Thermo Scientific unless specified otherwise. All primers were from Biomers.

### Barcoding and multiplexing

Polymerase chain reaction (PCR) products from all rounds of both *in vitro* selections were appended with specific hexanucleotide barcodes (5′-extensions to forward and reverse primers, see Supplementary Table S1) via PCR. PCRs were performed in 1 ml reaction scale (200 µl × 5) by adding purified PCR product from each selection round (1 ng/µl) to 1×-Hybrid PCR buffer (Roboklon), respective primers (0.5 µM), dNTP mix (0.2 mM, each) and Hybrid DNA Polymerase (0.02 U/µl, Roboklon). After three PCR cycles (denaturation: 30 s, 98°C; annealing: 30 s, 52°C; elongation: 30 s, 72°C), products were ethanol precipitated and further purified by PCR purification kit (Qiagen). Concentrations of purified barcoded PCR products were measured on Qubit 2.0 Fluorometer (Invitrogen), following manufacturer’s protocol, using the dsDNA HS Assay Kit (Invitrogen), and the barcoded PCR products of all rounds were mixed in equimolar concentrations (final volume: 0.34 ml, 5.5 ng/µl).

### Library preparation and NGS

Illumina sequencing libraries were prepared using the low-throughput protocol of the TrueSeq® ChIP Sample Preparation Kit. No size selection was performed after adapter ligation, and the number of PCR cycles was limited to 10. PhiX (10%) was added to the resulting library, which was sequenced on the MiSeq® System according to the manufacturer’s instructions (2 × 150 bp).

### Data treatment

The short reads resulting after sequencing were subjected to adapter trimming using the program Cutadapt (26). Quantification of sequence abundance and further analyses were done using custom *R*/Bioconductor scripts (27). To analyze only sequences with low error probability, all sequences that contained bases with Phred scores <20 (base-call probability <99%) at any position were excluded from further studies. Distinct sequences were quantified and sorted according to abundance.

### Multiple sequence alignment

Multiple sequence alignments were performed using Multalin (28) with Identity 1-0 symbol comparison table and parameters gap penalty 1, gap extension penalty 0.

### Categorization of DAse structures

Sequences were categorized as (i) typical DAse structures if they were able to form a structure similar to that of the minimized 49mer consensus-structure as reported in the original publication (6), containing three helices I, II, III and 5-nt upper bulge and a 6-nt lower bulge; (ii) optimal typical DAse structures if there were no mismatches in helices I, II and III [with the exception of certain mismatches in the closing base pairs of helix III, which have been previously shown not to decrease the catalytic activity (21)], if the upper bulge was UGCCA or UGCCG and if the lower bulge was AAUACU; (iii) typical DAse structures with suboptimal bulge if the upper bulge was not UGCCA or UGCCG, or if the lower bulge was not AAUACU; (iv) typical DAse structures with unstable helices if one of the helices I, II or III contained mismatches or bulges; and (v) energetically favored when the typical DAse structure appeared among the minimum free energy structures predicted by Mfold (29) without setting any constraints. Differences in free energy were estimated comparing the preferred structure predicted without constraints and the typical DAse secondary structure predicted with constraints.

### Validation of mutant MIRzyme reactivity—site-directed mutagenesis

To mutagenize region s8, RNA19 (3) template was amplified from a respective pCR2.1-plasmid (0.8 pg/µl) with primerA, respective mutprimers (Supplementary Table S2) and Hybrid DNA polymerase, as described above (in 50-µl scale). After 25 PCR cycles (denaturation: 30 s, 98°C; annealing/elongation: 1 min, 72°C), desired products were agarose gel purified. To mutagenize region s6, the plasmid encoding RNA19 was amplified by mixing plasmid (0.8 pg/µl) and 5′-monophosphorylated primers (primer s6-rev and the respective mutprimer, 500 nM each) dNTP mix (200 µM, each), 1× HF-buffer, dimethyl sulfoxide (4% v/v) and Phusion High Fidelity Polymerase (0.04 U/µl). After 25 PCR cycles (denaturation: 10 s, 98°C; annealing: 30 s, 66°C; elongation: 2 min, 72°C), linear double-stranded DNA was circularized by mixing 10 µl of PCR product, 1× T4 DNA ligase buffer and T4 DNA ligase (0.75 U/µl). After ligation (1 h, 22°C, 40 µl), 10 µl was transformed into *Escherichia coli* DH5α and plated on LB-agar (Sigma) with ampicillin. For each transformation, three clones were picked and cultured, and plasmids were isolated using the QIAprep Spin Miniprep Kit (Qiagen). Mutant transcription templates were prepared using Hybrid DNA polymerase, with plasmids mutated in s6 (4 pg/µl), or with gel-purified PCR products mutated in s8 (0.2 pg/µl), using primerA and primerB in 25 PCR cycles (same program as barcoding PCR). PCR products were purified via PCR-purification kit (Qiagen). Transcription templates were validated by sequencing (Seqlab).

### *In vitro* transcription and labeling

*In vitro* transcription of the non-mutated RNA19 and nine mutated versions of RNA19 was performed according to standard protocols (3). Polyacrylamide gel electrophoresis-purified RNA was radioactively labeled, with ^32^P-pCp (0.66 µM), as previously described (3).

### Reaction with the mechanistic inhibitor

To assess mutant reactivity, non-labeled RNA (10 µM), doped with radiolabeled RNA, was mixed with 1×- reaction buffer (300 mM NaCl, 200 mM KCl, 5 mM MgCl_2_, 5 μM MnCl_2_ and 5 μM CaCl_2_, pH 5.5). Biotin-PEG_4_-NH-D-Phe-Pro-Arg-chloromethylketone (mechanistic inhibitor; 800 µM) was added after renaturation (denaturation: 3 min, 80°C; renaturation: cooling to 10°C at a rate of 0.1°C/s, followed by 5 min, 10°C). After reaction (30 min, 25°C), RNA was ethanol precipitated. Product formation was measured as previously described (3), using streptavidin-based gel shift assays, visualized on a Typhoon 9400 (GE Healthcare) and quantified using the ImageQuant software (GE Healthcare). Product formation of mutants (average of three measurements) was normalized to that of RNA19 (100%).

## RESULTS

### Diversity analysis of DAse selection

For each pool (DNA template of the respective selection round), sequences were sorted according to read numbers (Materials and Methods, Supplementary Dataset S1) and at first, sequence diversity was analyzed for the DAse selection (Figure 1C, Supplementary Figure S2), taking into account the relative selection pressure (Supplementary Text S1). As the starting library was highly diverse (120 randomized positions–theoretical sequence space ∼10^72^; true complexity ∼10^14^), the initial rounds of the selection were too complex to be covered even by NGS (∼10^5^ reads per pool). The first slight enrichment was observed in pool 5 where nine sequences had >2 reads. The most prominent reduction in complexity was observed in pool 6 (when increased catalytic activity was detected first), and from pool 7 onward, the overall numeric pool diversity stagnated and remained constant although the catalytic activity still increased markedly under increased selection pressure (Figure 1B and C, Supplementary Figure S2, Supplementary Tables S3 and S4).

The sequencing data of the DAse ribozyme selection revealed an unexpectedly large reservoir of >25,000 distinct sequences in the final sequencing pool (out of ∼116,000 reads). Multiple sequence alignments identified 759 distinct groups of sequences in pool 7, compared with pool 10, which contained only 234 groups (Figure 1D and E, Supplementary Tables S5 and S6, see Supplementary Texts S2 and S3 for further details). In all, 197 groups were common to pools 7 and 10. In pool 10, these groups covered 97% of total reads and 87% of distinct sequences. Most sequences in pool 10 belonged to 33 groups with >100 members. Still, >3,400 sequences (3% total reads) did not belong to any group. Within each group, the major sequence covered, on average, ∼40% of total reads, and the other members differed from this major sequence, as observed in a recent aptamer selection (17), on average, by 2–3 mutations (see Supplementary Texts S2 and S3 for more details), different from sequencing errors (Supplementary Text S3, Supplementary Tables S7 and S8). Comparison of pools 7 and 10 revealed a decrease in structural diversity, compensated by an increase in diversity within the groups (Figure 1D and E, Supplementary Tables S5 and S6), which explains the stagnation of numeric diversity measures. Overall, the sequencing data indicate that >750 distinct RNA species with a catalytic activity above background were present in the starting library, a number that is much higher than generally anticipated for such a task (10,30).

### Evolution of the most abundant sequences toward highly active catalysts in the DAse ribozyme selection

**Figure 2.**
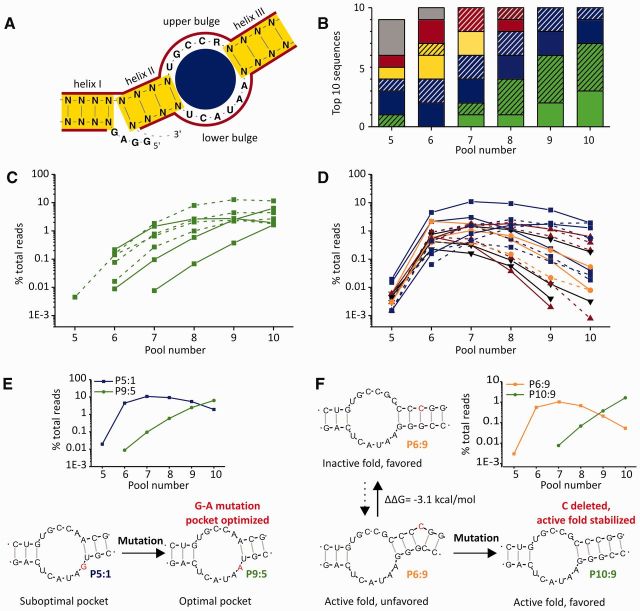
Sequence and structure evolution. (**A**) ‘Optimal’ typical DAse secondary structure and color-code for further panels. Blue: imperfect pocket, yellow: unstable helices, red: imperfect pocket and unstable helices, green: ‘optimal’ typical DAse, black/gray: no typical DAse fold. (**B**) Sequences found among the (maximum) 10 top ranking sequences in different pools, categorized by secondary structure prediction [hatched: energetically unpreferred, according to Mfold (29) secondary structure prediction]. (**C** and **D**) Abundance of all sequences found among the 10 top ranking sequences of each pool—either with ‘optimal’ typical DAse structure (**C**) or ‘suboptimal’ or atypical DAse structure (**D**), dashed lines represent energetically unpreferred typical DAse secondary folds. (**E**) Example of sequence evolution leading to optimization of the ribozyme’s catalytic pocket. (**F**) Example of sequence evolution leading to stabilization of an active fold.

We then analyzed the most abundant sequences (maximum 10) in each pool over the complete selection process (Figure 2A and B). To categorize them, four criteria were considered, based on knowledge from biochemical analyses of the major type of DAse ribozymes (6,21): (i) ability of forming a typical DAse secondary structure as that observed for the minimized 49mer, (ii) nucleotide composition of the catalytic pocket, (iii) stability of helices I, II and III and (iv) energetic favorability of forming a typical DAse secondary structure (for details see Materials and Methods). 

In pool 5, only one of the abundant sequences constituted a typical DAse catalyst with features optimal for catalytic activity in this type of ribozyme, whereas other ribozymes of this type had either suboptimal pockets or unstable helices. Three of the nine most enriched sequences of this pool could not be classified as typical DAses. More favorable typical DAse structures appeared in pool 7, after selection pressure was increased. Over the rounds of selection, the number of ‘optimal’ typical catalysts increased, whereas other ribozymes decreased. Focusing on individual top 10 sequences (Figure 2C and D, Supplementary Figure S3), the only species that showed high fitness (i.e., that increased in abundance) in the last pools, with increasing selection pressure, were the ‘optimal’ typical DAse ribozymes with perfect bulges and stable helices (Figure 2C). Among the top 10 sequences, ribozymes that contained a different bulge composition (1 nt difference with respect to ‘optimal’ typical DAse ribozymes) were found to have a reduced fitness, but were still better than those containing unstable helices or not assuming a typical DAse structure (Figure 2D, Supplementary Figure S3).

Among the typical DAse ribozymes, the ‘suboptimal’ ones were already observed in pool 5, where they dominated, whereas the majority of ‘optimal’ ones were first observed in pool 6 or 7. As ‘optimal’ typical DAse ribozymes have a much higher catalytic activity than most ‘suboptimal’ ones (21), they should have appeared first, had they been present in the starting library. This suggests their evolution from less efficient progenitor ribozyme sequences by mutation. Two illustrative examples of ribozyme evolution could be identified among the top ranking sequences. The first example is constituted by sequences P5:1 (pool 5’s top 1 sequence) and P9:5, which eventually outcompetes P5:1 (Figure 2E, Supplementary Figure S4). Both only differ by a single point mutation in the lower bulge of the catalytic pocket [P9:5: 5′-AAUACU-3′; P5:1: 5′-GAUACU-3′ - 5- to 10-fold lower activity (6,21)]. As it is extremely improbable (conditional probability: ∼10^-58 ^= true sequence diversity/theoretical sequence diversity = ∼10^14^/10^72^) that both sequences were present in the starting pool, P5:1 must have given rise to P9:5 by mutation. Another example for the improvement of fitness by mutation is the sequence pair P6:9–P10:9 (Figure 2F, Supplementary Figure S5). To test whether active catalysts generally evolved from less active precursors, we identified the major sequence of each of the 33 large sequence groups in pool 10, and traced back their origin to earlier generations (Supplementary Figure S6). According to the relative increase in abundance, we find that at least 15 of the 33 major sequences evolved from less active precursors by acquiring one or more mutations. Although most of these cases (among the most abundant sequences) represent typical DAse ribozymes, we observed the principle of evolution from precursors with lower fitness also for an atypical DAse ribozyme (Supplementary Figure S6D). The fitness of the evolved version in this group of ribozymes is comparable with that of many of the typical ones, showing that other efficient solutions to the problem of RNA catalyzing the Diels–Alder reaction exist in random space, but might be scarce.

### Structure-based analysis of DAse ribozyme evolution

**Figure 3.**
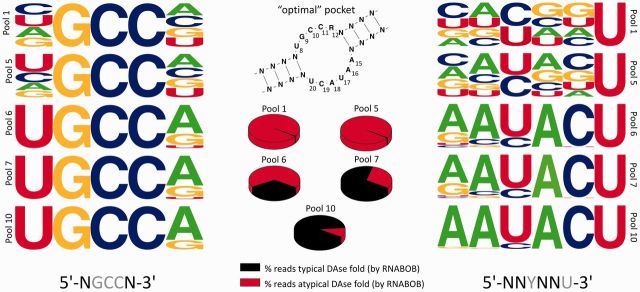
Evolution of upper and lower bulge in the typical DAse ribozymes. Nucleotide conservation obtained by NGS of DAse selection pools. To analyze the evolution of these bulges, several pools were analyzed for the nucleotide conservation in the upper and lower bulge (shown as frequency plots). The secondary structure is showing the preferred nucleotide composition of typical DAse ribozymes, pie-charts show the fractions of sequence reads that have a typical DAse fold identified by RNABOB and the sequences shown below the frequency plot show random (black) and (semi-)conserved (gray) nucleotides, as used in the RNABOB descriptor (see Supplementary Text S4).

Because functional RNAs are generally more conserved in structure than sequence, we studied the conservation and evolution of specific structural elements of the typical DAse ribozymes using RNABOB, a structure-based search tool (31). As indicated above, in the 49mer DAse ribozyme, the bulge regions are critical for catalysis (21). Applying a general descriptor (Supplementary Text S4, Supplementary Figures S7 and S8) of the typical DAse secondary structure to the sequence datasets from five different pools, bulge sequence element conservation was analyzed and visualized as position-specific nucleotide frequency plots (Figure 3). As expected, in pool 1, the bulges showed no conservation at positions that were kept variable in the descriptor (see Supplementary Text S4). In pool 5, in which few pool members had a typical DAse structure and catalytic activity was not detectable, only a slight shift toward preferred nucleotides was noted at certain positions. In pool 6, the percentage of typical DAse folds rose from 0.8 to 33%, and catalytic activity increased by a factor of 250, indicating that low evolutionary pressure primarily increased the abundance of typical DAse structures. At this point, however, larger changes in bulge composition became visible, with the ‘optimal’ nucleotides dominating at all positions. Pools 7 and 10 witness the evolutionary fine-tuning of the catalytic pocket, probably due to the high selection pressure. The ∼10^5^ reads representing typical DAse ribozymes collected by the descriptor in pool 10 contained almost exclusively the ‘optimal’ nucleotides at each position, tolerating only mutations with ≥35% relative activity (Supplementary Figure S9). Selection and evolution have thus led to a strong bias for ‘optimal’ typical DAse structures in the final pool. 

### Evolution in the MIRzyme selection and rational improvement of catalysts based on NGS analysis

**Figure 4.**
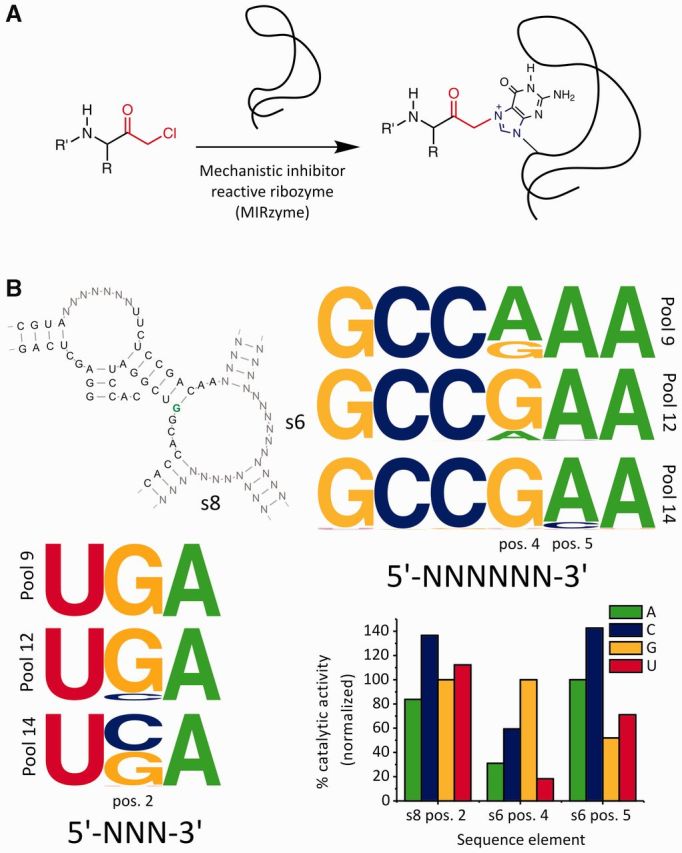
Conservation of the sequence in structural elements. (**A**) Reaction scheme showing the reaction catalyzed by MIRzymes. (**B**) Conserved four-way junction structure of a group of highly active MIRzymes (with reaction site highlighted in green). Frequency plots showing the sequence conservation of two single-stranded bulge regions (s6 and s8) in the conserved four-way junction structure, analyzed using RNABOB, as described in Supplementary Text S6. The bar-graph depicts normalized reactivity for all RNA19 mutants, relative to RNA19 (average of triplicate, S.D. <5% of average wild-type activity, except s6, position 5 A to U – 10.8%).

Because the NGS data analysis for DAse selection revealed a good correlation between the evolution of sequence elements and known biochemical data (see Figure 3 and Supplementary Figure S9), we argued that it should be possible to predict the biochemical behavior for less characterized ribozymes from a different selection based on NGS data, and to validate such predictions. Therefore, we also analyzed the recent MIRzyme selection [Figure 4A, (3)] by NGS. Similar to the DAse ribozymes, we observed a strong correlation between initial decreases in numeric diversity and increases in catalytic activity (Supplementary Figure S1, Supplementary Tables S9 and S10, Supplementary Text S5). Furthermore, as for the DAse ribozymes, we observed examples of evolution by mutation (mainly substitutions, Supplementary Figure S10 and deletions, Supplementary Figure S11), which led to the optimization of catalytically active ribozyme structures. Earlier analyses of a small number of isolated sequences revealed that the most active MIRzymes form a conserved four-way stem-loop structure (3). Here, similar to the DAse analysis, RNABOB was used to study the evolution of structural elements in MIRzymes that share this conserved structure. Specific descriptors (Supplementary Text S6 and Supplementary Figure S12) were applied on the sequence datasets of three different pools, pool 9 [where a first significant amount of active RNAs appeared, Supplementary Figure S1, (3)], pool 12 (pool resulting from the first round with increased selection pressure) and the last pool (pool 14). The position-specific conservation was analyzed and was visualized as frequency plots (Figure 4B). 

In the single-stranded bulge region s6, changes in nucleotide frequency were observed at positions 4 and 5, whereas the other four positions were conserved in all three rounds. At position 4, the initially dominating nucleotide A was gradually replaced by G in pools 12 and 14, where selection pressure was increased. At position 5, A was dominant in pool 9 but C appeared in a small fraction of sequences after the application of higher selection pressure in pool 12 and increased in frequency in pool 14. Observing that certain changes were favored and others disfavored by evolution under high selection pressure, we predicted that a mutation from G to A at position 4 would result in reduced, but measurable, catalytic activity, whereas an A to C mutation at position 5 should lead to increased activity.

In the case of the single-stranded bulge region s8, 5′-UGA-3′ was conserved in pool 9. Under higher selection pressure, a slight occurrence of C was observed at position 2, and C was about equally represented as G in pool 14. Therefore, we predicted that a G to C mutation at this position should lead to increased reactivity.

To confirm these predictions, the three respective positions were subjected to site-directed mutagenesis, creating altogether nine mutants of the wild-type (RNA19) (3), which shared the composition that dominated in pool 14. For all mutants, relative catalytic activity was measured (Figure 4B). We found that mutating position 4 of s6 to A resulted in a marked decrease to only 31% of wild-type activity. In contrast, as predicted, mutating position 5 of s6 to C resulted in an increase to ∼143% of wild-type activity. Mutating position 2 of s8 to C resulted in a similar increase to ∼137%. A slight increase in catalytic activity (to ∼112%) was observed when mutating the same position to U, which correlates with the appearance of the nucleotide U (in a minute proportion) at this position in pool 14.

Thus, we could show that, unlike in the typical DAse ribozymes, the dominating sequence composition in the final round of selection (the sequence composition shared by the wild-type RNA19) did not represent the optimal composition for this type of MIRzyme. In the MIRzyme selection, other compositions, which allow higher catalytic activity, are still developing during the later rounds of selection and are slowly emerging in the selection pools, showing that evolution is still ongoing in this selection experiment. Observing changes in the nucleotide frequency, it was possible to predict nucleotide substitutions that improved catalytic activity.

This example shows that in the case of ongoing evolution (as observed for the MIRzyme), the data acquired in NGS analysis can be used for the rational design of improved catalysts, even before the best possible variants of the ribozyme dominate the selection pools.

## DISCUSSION

Our study shows that *in vitro* selection of ribozymes from long randomized pools (at least in the range of the 120 and 170 randomized positions, of our respective examples) relies on evolution and not just on the selection for highest catalytic activities. A considerable proportion of highly active ribozymes was shown to have evolved from suboptimal precursors by acquiring at least one mutation that led to either stabilization of the active fold or optimization of the catalytic pocket. Covering only a minute fraction of theoretical sequence space in the starting library, the role of mutations is relevant for any ribozymes evolving from libraries too large (in length) to cover the complete sequence space. Imperfect ribozymes—which are much more abundant in random space than perfect ones—can serve as precursors for further evolution, and improved catalysts will be preferred over the selection, especially under increased selection pressure.

Spontaneous mutational events during transcription, reverse transcription and PCR [estimated error rates: 1 in 10^4^–10^5^ incorporated nucleotides (32–35)] are thus of utmost importance for the exploration of a larger fraction of sequence space than covered by the starting library. Thus, the use of non-proofreading enzymes in selections is critical for the efficient evolution of sequence pools. For sequences of the lengths used here, ∼1 in 10 to 1 in 100 sequences will acquire a mutation in each round. Because most mutations will not cause a gain of function, the number of improved catalysts will be much smaller than the number of progenitor copies and non-improved mutants. Consequentially, error rates that allow more mutations (e.g., mutagenic PCR) could accelerate the evolutionary process as long as mutation rates are not exceedingly high, in which case active sequences might not be retained any longer.

Selections are generally started at low selection pressure to avoid the loss of the initially scarce active sequences. According to our study, this also helps in avoiding the loss of progenitor ribozymes with low activity. In order for resulting (improved) catalysts to outcompete the progenitor sequences, a sufficient number of selection rounds and a timely increase of selection pressure are of use.

As a methodological aspect, we also show that NGS data can be used to identify nucleotides, which can be mutated to rationally improve existing selected catalysts, even when the best possible catalysts do not yet dominate the selection pool. This makes NGS an interesting tool for both, studying the evolution of ribozymes and improving them rationally.

Our findings not only substantiate previous assumptions about the basic principles underlying the *in vitro* selection of ribozymes, but may also help improving the outcome of future selections by preserving more precursors and allowing their accelerated evolution into highly active catalysts, as well as their rational improvement with the help of NGS data analysis.

## SUPPLEMENTARY DATA

Supplementary Data are available here.

## FUNDING

Hartmut Hoffmann-Berling International Graduate School of Molecular and Cellular Biology (HBIGS) (to M.-L.W.); Deutsche Forschungsgemeinschaft [Ja 794/3-5, SFB 623]. Funding for open access charge: Institutional budget.

*Conflict of interest statement*. None declared.

## Supplementary Material

Supplementary Data
