# Supplementary material for: Next-generation sequencing reveals how RNA catalysts evolve from random space
Source: Nucleic Acids Res. 2013 Oct 23;42(2):1303–10. doi: 10.1093/nar/gkt949 (PMC3902939; doi:10.1093/nar/gkt949)
Supplement: Supplementary Data [file gkt949_suppmat.zip › nar-02361-z-2013-File006.pdf]

Supplementary Information

Next-generation sequencing reveals how RNA catalysts evolve from random space

Sandeep Ameta[1], Marie-Luise Winz[1], Christopher Previti, Andres Jäschke[*]

[1]These authors contributed equally to this work.

[*]Corresponding author. E-mail: jaeschke@uni-hd.de

Table of contents

Supplementary Text

S1. Relative selection pressure and selection conditions of the DAse selection. 2
S2. Detailed study of the diversity in DAse selection pools 7 and 10. 2
S3. Analysis of mutations and differentiation from sequencing errors. 3
S4. Structure-based analysis of DAse pools using RNABOB. 5
S5. Relative selection pressure and selection conditions of the MIRzyme selection. 5
S6. Structure-based analysis of MIRzyme pools using RNABOB. 6

Supplementary Figures

S1. MIRzyme selection conditions and different measures of diversity in the MIRzyme selection pools. 7
S2. Different measures of diversity in the DAse selection pools. 8
S3. Evolution of DAse selection pools. 9
S4. Optimization of the catalytic pocket by evolution. 10
S5. Another example of DAse ribozyme evolution. 11
S6. 10 representative examples of the evolution of dominant sequences from the 33 large groups of pool 10. 12
S7. Typical DAse secondary structure with denotation as in the RNABOB descriptors. 13
S8. Percentage of total reads identified by the different RNABOB descriptors in the different DAse pools. 14
S9. Biochemical analysis data of 49mer DAse. 15
S10. Example of MIRzyme evolution by substitution. 16
S11. Example of MIRzyme evolution by deletion. 17
S12. MIRzyme secondary structure with denotation as in the RNABOB descriptors. 18

Supplementary Tables

S1. Barcoding primers. 18
S2. Primers for site-directed mutagenesis. 19
S3. Pool diversity in the DAse selection by total reads. 20
S4. Pool diversity in the DAse selection by distinct sequences. 21
S5. Overview of different groups identified in the DAse selection pool 7 by multiple alignments. 22
S6. Overview of different groups identified in the DAse selection pool 10 by multiple alignments. 23
S7. Average percentage of each error type among 23 groups of DAse ribozymes. 24
S8. Average percentage of the two different types of transitions and transversions, as well as of indel-type errors. 25
S9. Pool diversity in the MIRzyme selection by total reads. 26
S10. Pool diversity in the MIRzyme selection by distinct sequences. 27

Supplementary Data for this manuscript also include the following:

Supplementary Dataset 1 as excel file.

Supplementary Text

S1. Relative selection pressure and selection conditions of the DAse selection. The relative selection pressure was calculated as a multiplication of fold-decrease in the substrate concentration (biotin maleimide) and the incubation time w.r.t. the starting reaction conditions. For example, relative selection pressure in round 6 is 60, i.e., selection pressure due to decrease in time of incubation: 60 min to 5 min (12-fold decrease) and selection pressure due to decrease in concentration of substrate: 25 µM to 5 µM (5-fold decrease). These reaction conditions are taken from the original DAse selection paper (6) after removing the rounds that were discontinued in the original selection (original rounds number 6 and 7). The original rounds number 6a and 7a are renamed as 6 and 7, respectively, in the current study. The details of the incubation time and substrate concentration in each round are compiled in the following table.

The reader should note that the DNA pools analyzed in this study are numbered according to the round for which they constituted the transcription template. However, the results of selection are seen in the DNA pool resulting from the selection round only (e.g., the result of increased selection pressure in round 6 can be seen only in the pool following round 6, namely pool 7).

Round	Template for this round	Time (min)	c (biotin maleimide, µM)	Relative selection pressure
**1**	pool 1	60	25	1
**2**	pool 2	60	25	1
**3**	pool 3	60	25	1
**4**	pool 4	60	25	1
**5**	pool 5	60	25	1
**6**	pool 6	5	5	60
**7**	pool 7	1.5	2.5	400
**8**	pool 8	1	2.5	600
**9**	pool 9	1	2.5	600
**10**	pool 10	1	2.5	600

S2. Detailed study of the diversity in DAse selection pools 7 and 10. In order to determine the nature of variation (either completely different sequences or variants of common sequences generated due to substitutions, deletions and insertions - or sequencing errors), we separated all sequences of pool 7 and pool 10 into sequence groups using multiple sequence alignments (Supplementary Tables S5, S6). Both pools had similar numeric diversity and total read numbers (Supplementary Tables S3, S4). This analysis resulted in 759 sequence groups in pool 7 and 234 sequence groups in pool 10, each group containing between 2 and > 1000 members.

Levenshtein (or edit) distances between groups were ≥ 50, meaning that at least 50, and mostly more substitutions, deletions and insertions would be necessary to turn one sequence into the other. Mismatches between sequences from different groups were distributed along the complete sequences. Levenshtein distances within the groups were generally < 8, and mostly comprised between 1 and 4 (see Supplementary Text S5 for more detailed analysis of mutations in 23 of the groups from pool 10), although, in the case of group members carrying longer deletions, large Levenshtein distances (occasionally > 50) were also observed. In these cases, however, one or two longer stretches of nucleotides were deleted, compared to the consensus sequence of the group, whereas the remaining parts of the sequence only contained few or no mismatches.

In pool 7, around 75% of total reads (51% of distinct sequences) are covered by groups with > 100 members. Out of those, the major two groups with > 1000 reads cover 25% of total reads (25% of

distinct sequences). Still, 20% of reads (28% of distinct sequences) were attributed to groups with ≤ 100 members. 5690 distinct sequences (5876 reads), covering 5% of total reads (20% of distinct sequences) could not be attributed to any other sequence from the pool, and are thus unique. At least 433 (~8%) of these sequences have typical DAse features, so they are likely to have catalytic activity. Among the sequence groups, at least 390 have typical DAse features. These are particularly common (> 75%) among the larger groups, and less common (≤ 60%) among the smaller groups with < 100 members.

In pool 10, around 93% of total reads (87% of distinct sequences) are covered by groups with > 100 members. Out of those, the major five groups cover > 50% of total reads (> 40% of distinct sequences), showing a shift towards less and larger groups. Only a minor fraction of sequences were found in groups with ≤ 100 members. 3430 distinct sequences (3462 reads), covering 3% of total reads (13% of distinct sequences), could not be aligned to any other sequence from this pool, and are thus unique. At least 152 (~4%) of these sequences are likely to be catalytically active since they have typical DAse features. Compared to pool 7, groups with typical DAse features are more common in pool 10. Here, the large groups consist mainly of typical DAse sequences (≥ 89%). Although still less common in the smaller families, the typical DAse sequences make up ≥ 49% of these smaller families.

Pool 7 and pool 10 have 197 groups in common. 12 unique sequences from pool 7 are represented as groups in pool 10. The remaining groups (only small ones) were not sampled in pool 7, yet. Comparing groups in pool 7 and pool 10, few groups gained in importance (ranking much higher in the later pool), whereas certain groups ranked lower in pool 10. Most groups, however, were lost, and were not sampled any longer in pool 10.

Altogether, the comparison of diversity in pools 7 and 10 revealed a loss in structural diversity, which was compensated by a growing number of members in certain other families. This loss affected atypical DAse ribozyme families more than the typical ones, showing an increasing preference of the typical DAse fold under increased selection pressure.

S3. Analysis of mutations and differentiation from sequencing errors. To elucidate whether the sequences of each group are true variants that may have arisen during the selection procedure due to errors in transcription and reverse transcription-PCR (RT-PCR) or whether the majority of variants arose from sequencing errors, we performed group-specific multiple sequence alignments (Multalin (28), see Materials and Methods). The outputs were manually corrected, and the number of different mutations, deletions and insertions w.r.t. the consensus sequence was calculated (without taking into account copy numbers).

Analyzing the sequences belonging to about 87% of total reads (from 23 groups), we found all 12 possible point mutations (substitutions), as well as insertions and deletions, however, distributed with certain preferences, which varied slightly among the groups. For true mutations, this can be explained by selective advantages, which may arise from diverse mutations that vary by the group. We calculated averages for each possible mutation within each group and among all 23 groups, to determine the percentage of each error among all sequences (Supplementary Tables S7, S8). Altogether, the most preferred mutations were the transitions of C to T (19%) and A to G (19%), followed by T to C (14%) and G to A (8%). Compared to transitions, transversions were very unfavored. Insertions (8%) and deletions (14%) were also common. The number of insertion and deletion events may be overestimated since each nucleotide that was deleted or inserted was counted separately.

To verify that those errors are genuine mutations, we compared these data to studies concerning sequencing errors of Illumina sequencing methods. In Illumina sequencing, insertions and deletions (indels) were found to be very rare (36), and transitions were not more common than other base call errors, accounting for about 23 to 33% of Illumina sequencing errors (36,37). On the other hand, about 79 to 100% of mutations induced by *Taq* polymerase were found to be transitions (38-40). Since indel errors account for 23% of errors in our study, and transitions account for 60% of total mismatches (or 78% of non-indel errors - total transitions / total point mutations; 60% / 77%), we conclude that most of the diversity in our selection pool is not due to sequencing errors, but rather to mutations.

A bias towards transversions of purine to pyrimidine (R to Y) over pyrimidine to purine (Y to R) was noted, which indicates strand-specific mutagenesis. During the PCR in the selection, only the 2nd strand formation allows for strand-specific mutagenesis, since both strands should have the same probability of mutation in further cycles, which would lead to a balance of transversions from R to Y and vice-versa. However, the 2nd strand synthesis is not the only strand-sensitive event; errors can also be introduced during transcription, which would explain the bias. A bias towards the transitions A to G and C to T was also noted. Interestingly, *Taq* polymerase favors A to G transitions over G to A, but also T to C transitions over C to T transitions (38-40). We observed the same preference for the purines, but the opposite for the pyrimidines. T7 RNA polymerase, which also contributes to amplification and therefore to mutation (error rates of *Taq* and T7 RNA polymerase are comparable) prefers A to G mutations, like *Taq* polymerase, but seems to favor C to U over U to C mutations (33), reflecting the wobble base-pairing potential of G and U.

Furthermore, to quantify how many mutations were acquired during the selection process, we calculated the Levenshtein distance, or edit distance. The Levenshtein distance indicates the minimum number of point-mutations, deletions and insertions needed to convert one sequence into another. When the average Levenshtein distance of members within each of the 23 groups covering 87% of reads was calculated w.r.t. the consensus sequence, the lowest overall Levenshtein distance was 0 (no mutation), and the highest was 74, in a sequence with a long insertion. The lowest average Levenshtein distance within a group was found to be 1.7, while the highest was 6.2 (in a group that contained many members with deletions). The average of average Levenshtein distances was 2.5 (± 0.9), indicating that each distinct sequence of a group has acquired 2-3 sequence changes on average after 9 rounds of evolution. (While, in general, point mutations should happen independently, insertions and deletions of multiple nucleotides can happen in a single event, so that the calculated Levenshtein distance might overestimate the mutational events slightly).

Interpreting these results, it should be kept in mind that the selection pools were subject to selection pressure, so that the mutation rates we found could be biased, and might not represent the combined mutation rates of all enzymes used in this study in an exact way. In comparison, Zimmermann *et al.* studied the mutations occurring in a neutral genomic SELEX process, where no selection pressure was applied at all. There, the authors found an increasing A, and a decreasing G content, as well as a weak trend towards increasing U content (41). However, since a different DNA polymerase (*Pfu*) and different numbers of amplification cycles were employed, the data are not directly comparable.

Altogether, our data imply that the biggest proportion of diversity in the DAse selection is reached by the variation of certain major catalytically active sequences. These mutations help exploiting the local sequence (and sometimes structure) space around certain sequences.

S4. Structure-based analysis of DAse pools using RNABOB (31). For typical DAse ribozymes, the descriptors were designed according to the biochemical activity information from one earlier study (see reference 21), i.e. nucleotides which were essential for the catalytic activity were kept conserved, since ribozymes mutated at these positions would very likely be inactive or have an extremely low activity. Initially, three similar descriptors (which differ only in the number of mismatches allowed in the shaded regions, see below) were used and the total sequence reads from each round were analyzed (see Supplementary Figures S7, S8). Descriptor I was found to be too strict, as only ~30% sequences were picked up in pool 10. On the other hand, descriptor III was the most sensitive, and allowed us to pick up a maximum of total reads, but turned out to be less specific than the other descriptors, as it was also picking up >15% of total reads in pool 1, where the active catalysts should be far below 1%. Descriptor II was found to be the best compromise between sensitivity and specificity. This descriptor was then used to study the sequence element conservation in the structure.

Outputs from RNABOB descriptor II were used to analyze the position specific sequence conservation in RNA structure elements using frequency plots from WebLogo (42). To create frequency plots, all sequence combinations of a sequence element were identified, sorted and submitted to the web-server based WebLogo program, taking into account their % occurrence in total reads.

```
# descriptor I

s1 h1 s2 h1' h2 s3 h3 s4 h3' s5 h2' s6

s1 0 GGAG
h1 0:1 CTCNN**:**NNGAG
s2 0 NNNN[77]
h2 0:0 NNNR:YNNN
s3 0 NGCCN
h3 0:0 NNNN:NNNN
s4 0 NNNN[77]
s5 0 NNYNNT
s6 0 NNNN[150]
```

```
# descriptor II

s1 h1 s2 h1' h2 s3 h3 s4 h3' s5 h2' s6

s1 0 GGAG
h1 0:2 CTCNN**:**NNGAG
s2 0 NNNN[77]
h2 0:0 NNNR:YNNN
s3 0 NGCCN
h3 0:2 NNNN:NNNN
s4 0 NNNN[77]
s5 0 NNYNNT
s6 0 NNNN[150]
```

```
# descriptor III

s1 h1 s2 h1' h2 s3 h3 s4 h3' s5 h2' s6

s1 0 GGAG
h1 0:3 CTCNN**:**NNGAG
s2 0 NNNN[77]
h2 0:2 NNNR:YNNN
s3 0 NGCCN
h3 0:2 NNNN:NNNN
s4 0 NNNN[77]
s5 0 NNYNNT
s6 0 NNNN[150]
```

S5. Relative selection pressure and selection conditions of the MIRzyme selection. The relative selection pressure was calculated as a multiplication of fold-decrease in the substrate concentration (biotin PPACK) and the incubation time w.r.t. the starting reaction conditions. These reaction conditions are taken from the original MIRzyme selection paper (3). The details of the incubation time and substrate concentration in each round are compiled in the following table.

DNA pools analyzed in this study are numbered according to the round for which they constituted the transcription template. The result of increased selection pressure in round 11 is only visible in the resulting pool, pool 12. Pool 14 was not used for a 14th round of selection.

Round	Template for this round	Time (min)	c (biotin PPACK, µM)	Relative selection pressure
**1**	pool 1	120	750	1
**2**	pool 2	120	750	1
**3**	pool 3	120	750	1
**4**	pool 4	120	750	1
**5**	pool 5	120	750	1
**6**	pool 6	120	750	1
**7**	pool 7	120	750	1
**8**	pool 8	120	750	1
**9**	pool 9	120	750	1
**10**	pool 10	120	750	1
**11**	pool 11	24	150	25
**12**	pool 12	24	150	25
**13**	pool 13	24	150	25
**14**	pool 14	-	-	-

S6. Structure-based analysis of MIRzyme pools using RNABOB (31).

In the case of MIRzyme, family-specific descriptors were used (see below and Supplementary Figure S12). These families were the same as those mentioned in the original selection paper (3). The outputs from these family-specific descriptors were combined to create frequency plots and predict the conservation. The length of the respective sequence elements was not equal in all four families. Therefore, to simplify the analysis, for element 's6' outputs from descriptors for family I, II and III were combined and for sequence element 's8' outputs from descriptors from family I and III were combined (as in the case of family II the element s8 was sometimes found to be only 2 nucleotides long).

```
# MIRzyme family I

h1 s1 h2 s2 h2' s3 h3 s4 h4 s5 h4' s6 h5 s7 h5' s8 h6 s9 h6' s10 h3' h1'

h1 0:0 GGA:UCC
s1 0 GC
h2 0:2 UCAGC**:**GCUGA
s2 0 N[7]N
s3 0 N[10]N
h3 0:1 UCCGAC:GUCGGA
s4 0 NN
h4 0:1 NNNN:NNNN
s5 0 N[120]N
s6 0 NNNNNN
h5 0:1 NNNN:NNNN
s7 0 N[22]N
s8 0 NNN
h6 0:1 GGUG**:**CACC
s9 0 N[5]N
s10 0 ACG
```

```
# MIRzyme family II

h1 s1 h2 s2 h2' s3 h3 s4 h4 s5 h4' s6 h5 s7 h5' s8 h6 s9 h6' s10 h3' h1'

h1 0:0 GGA:UCC
s1 0 GC
h2 0:2 UCAGCC:GGCUGA
s2 0 N[70]N
s3 0 N[9]N
h3 0:1 UCCGAC:GUCGGA
s4 0 NN
h4 0:1 NNN:NNN
s5 0 N[35]N
s6 0 NNNNNN
h5 0:1 NNN:NNN
s7 0 N[22]N
s8 0 NN
h6 0:1 GGUG**:**CACC
s9 0 N[34]N
s10 0 ACG
```

```
# MIRzyme family III

h1 s1 h2 s2 h2' s3 h3 s4 h4 s5 h4' s6 h5 s7 h5' s8 h6 s9 h6' s10 h3' h1'

h1 0:0 GGA:UCC
s1 0 GC
h2 0:2 UCA*C:GC*GA
s2 0 N[26]N
s3 0 N[9]N
h3 0:1 UCCGAC:GUCGGA
s4 0 NN
h4 0:1 NNN:NNN
s5 0 N[8]N
s6 0 NNNNNN
h5 0:1 NNN:NNN
s7 0 N[20]N
s8 0 NNN
h6 0:1 GGUG*:*CACC
s9 0 N[104]N
s10 0 ACG
```

Supplementary Figures

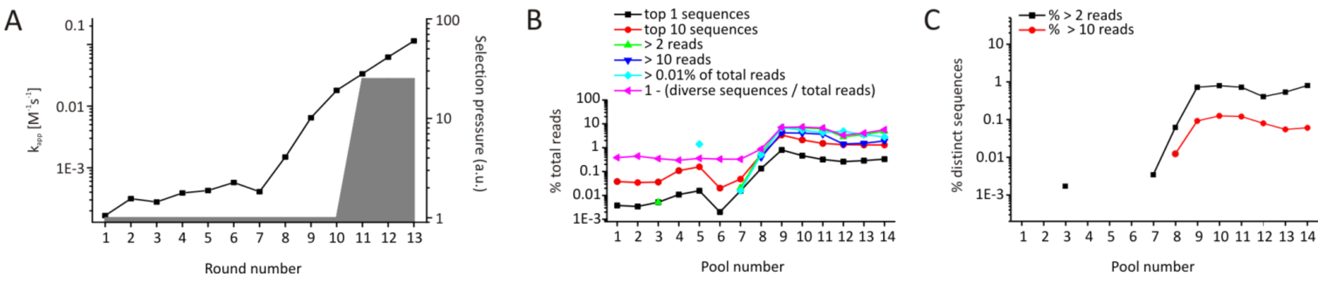

Supplementary Figure S1. MIRzyme selection conditions and different measures of diversity in the MIRzyme selection pools. (**A**) Selection profile with apparent rate constant k_{app} and relative selection pressure, taken from the original publication (3), (**B**) Percentage of total reads represented by the most abundant sequence (top 1), the 10 most abundant sequences (top 10), sequences with > 2 reads, sequences with > 10 reads, sequences with > 0.01% of total reads and 1 - (distinct sequences / total reads). (**C**) Percentage of distinct sequences represented by sequences with > 2 reads or > 10 reads. Comparing the development of k_{app} (**A**) and numeric sequence diversity or pool complexity (**B**, **C**), the most significant reduction in pool complexity is observed between pools 7 and 9. Corresponding to this development, the largest differences in k_{app} are noted between rounds 7 and 9. However, k_{app} still rises in the later rounds, whereas the numeric diversity stagnates after pool 9.

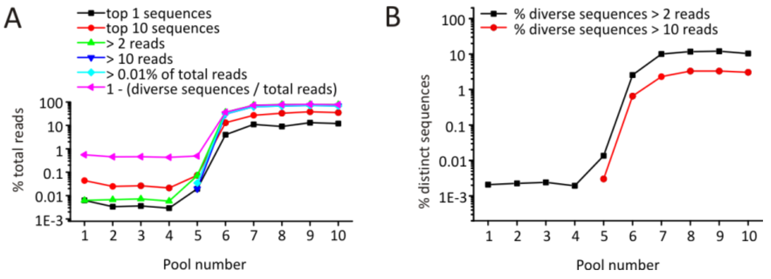

Supplementary Figure S2. Different measures of diversity in the DAse selection pools. (**A**) Percentage of total reads represented by the most abundant sequence (top 1), the 10 most abundant sequences (top 10), sequences with > 2 reads, sequences with > 10 reads, sequences with > 0.01% of total reads and 1 - (distinct sequences / total reads). (**B**) Percentage of distinct sequences represented by sequences with > 2 reads or > 10 reads.

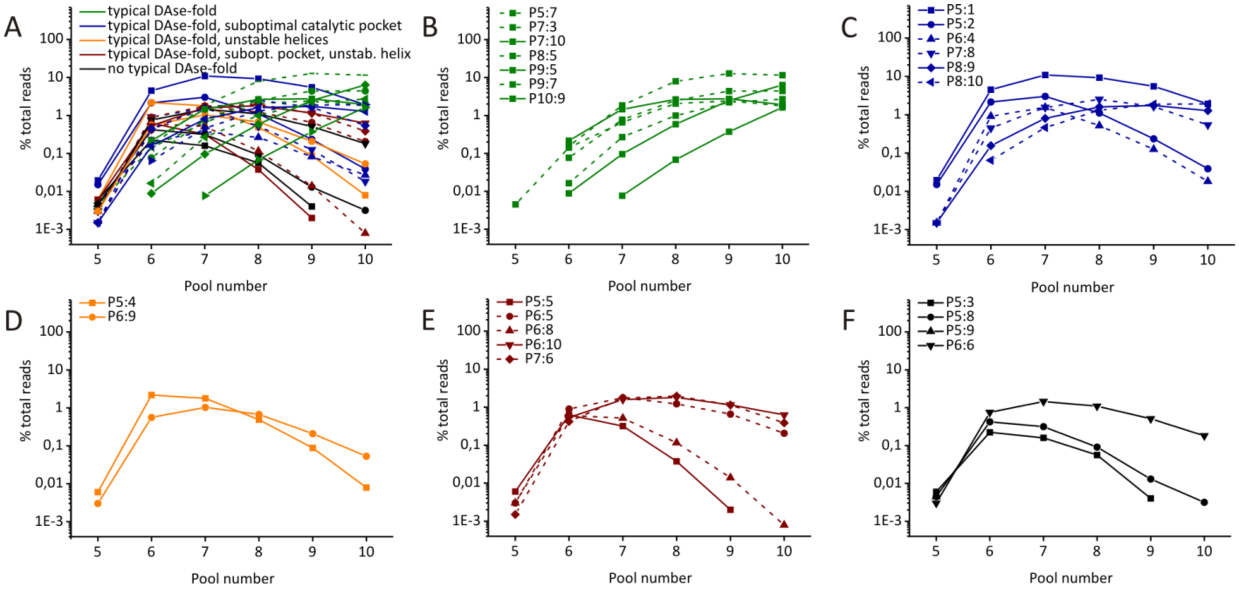

Supplementary Figure S3. Evolution of DAse selection pools. The abundance of different categories of DAse ribozymes is plotted as percentage of total reads. (**A**) all categories, (**B**) “optimal” typical DAse ribozymes, (**C**) typical DAse ribozymes with suboptimal catalytic pocket composition, (**D**) typical DAse ribozymes with unstable helices, (**E**) typical DAse ribozymes with suboptimal catalytic pocket composition and unstable helices, (**F**) atypical DAse ribozymes. Dashed lines represent energetically unfavored typical DAse folds.

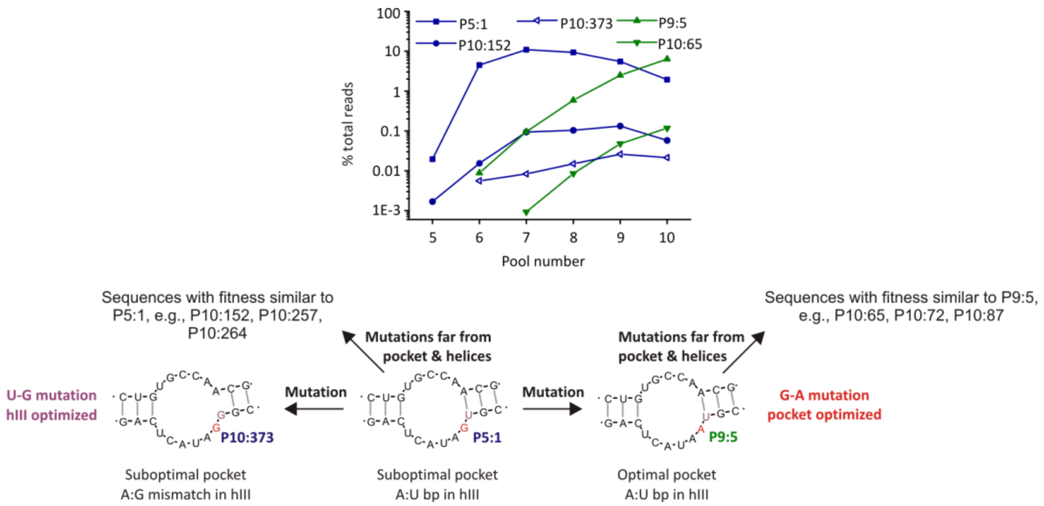

Supplementary Figure S4. Optimization of the catalytic pocket by evolution. While a mutation from G to A in the DAse lower bulge leads to an optimization of the catalytic pocket, which is reflected by a steeper increase in abundance (higher fitness), mutations that are situated far from the catalytic pocket and helices I, II, III do not influence the fitness of the ribozymes (compare P5:1 to P10:152, or P9:5 to P10:65). Although only one example is shown, we detected several examples of multiple mutants with both, perfect or imperfect pocket composition, with a fitness very similar to the respective examples shown here. A mutation in helix III from U to G causes an A:G mismatch in the closing bp, which increases catalytic activity by about 20% (21). This is reflected by slightly higher fitness of P10:373, compared to P5:1 or P10:152. An example of an optimized pocket in combination with an optimized helix III was also found in the NGS data, but read numbers were too low to evaluate its fitness.

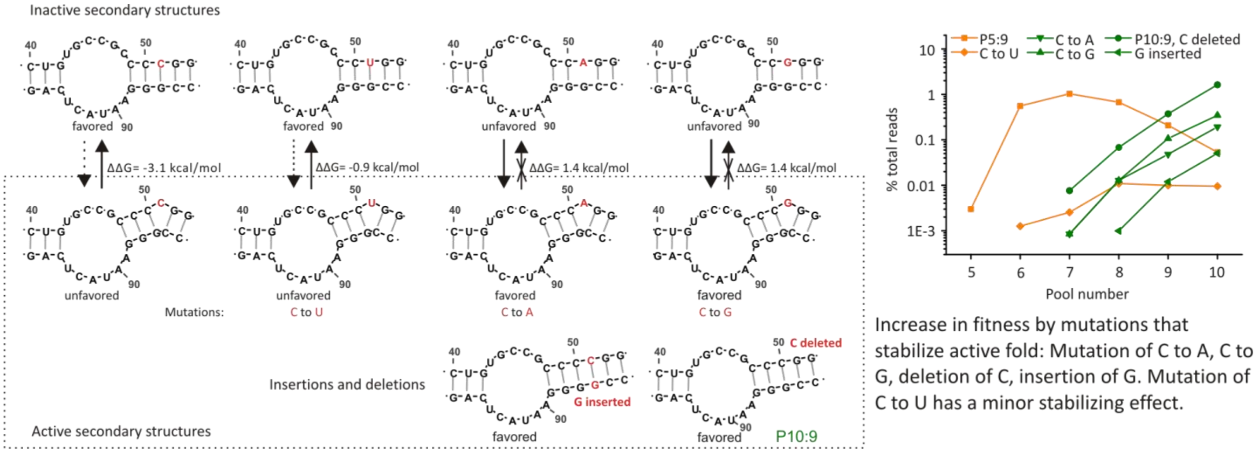

Supplementary Figure S5. Another example of DAse ribozyme evolution. Evolution from an unstable helix III in the active fold (orange in graph) to a stable helix III in the active fold (green in graph). P6:9 (upper left) and P10:9 (lower right) differ by the presence of a C at the 3'-end of the catalytic pocket upper bulge (in P6:9) that destabilizes the active fold (unstable helix III in active fold) and causes a preference for an alternative, inactive fold. The transition from active to inactive fold results in a difference in free energy of ~-3 kcal/mol. In the absence of this nucleotide, P10:9 can fold into an "optimal" typical DAse structure and has a higher fitness. Other variants of P6:9 were observed in the NGS data. While the mutation of the additional C to U leads only to a minor increase in fitness, (inactive fold is still preferred by about 0.9 kcal/mol), the mutation of this C to A or G increases the fitness (active fold is now preferred by about 1.4 kcal/mol). An insertion of a G that can pair with the additional C increases the fitness similar to the deletion or mutation of C. For most mutations, we were able to detect several examples with very similar fitness. However, for simplicity, only one representative example of each is shown. Secondary structures based on prediction by Mfold (29).

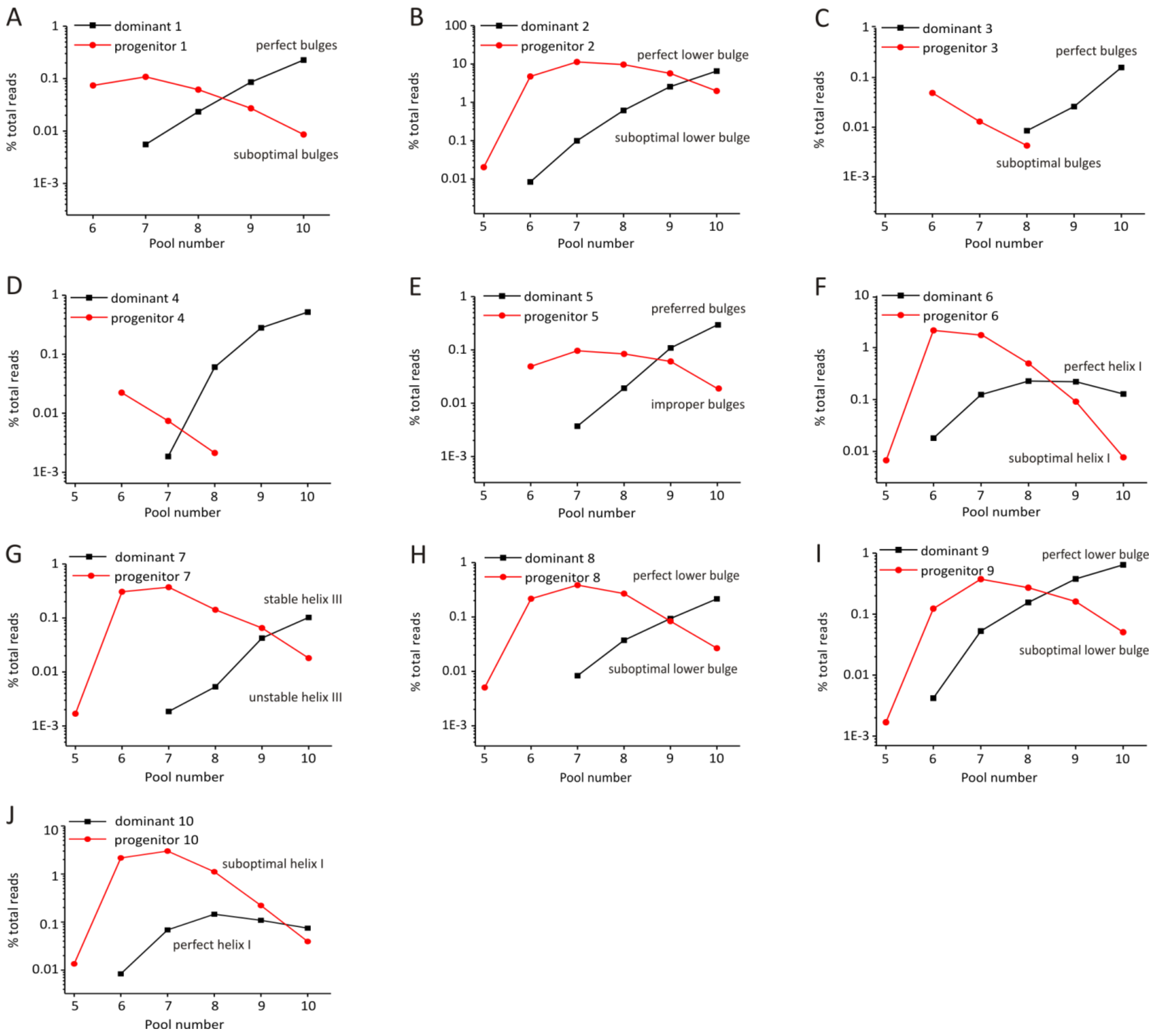

Supplementary Figure S6. 10 representative examples of the evolution of dominant sequences from the 33 large groups of pool 10. Relative abundance of the dominant sequences and their progenitor sequences (of lower fitness) are shown (**A** to **J**). These 33 groups contain > 100 members each. The dominant and progenitor sequence differ from each other by 1-2 point mutations in catalytically important regions (catalytic pocket or helices I, II, III). Dominant and progenitor sequences 4 (**D**) do not have a typical DAse fold. The dominant sequence has been identified previously by cloning and has been found to have high catalytic activity of k_{app} = 190±8 (43). The fitness of dominant sequence 4 is comparable to that of many "optimal" typical DAse ribozymes.

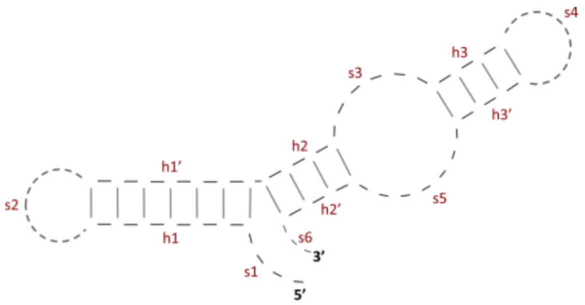

Supplementary Figure S7. Typical DAse secondary structure with denotation as in the RNABOB descriptors.

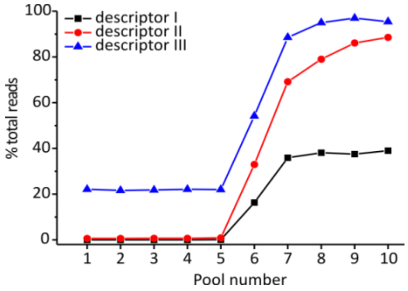

Supplementary Figure S8. Percentage of total reads identified by the different RNABOB descriptors in the different DAse pools.

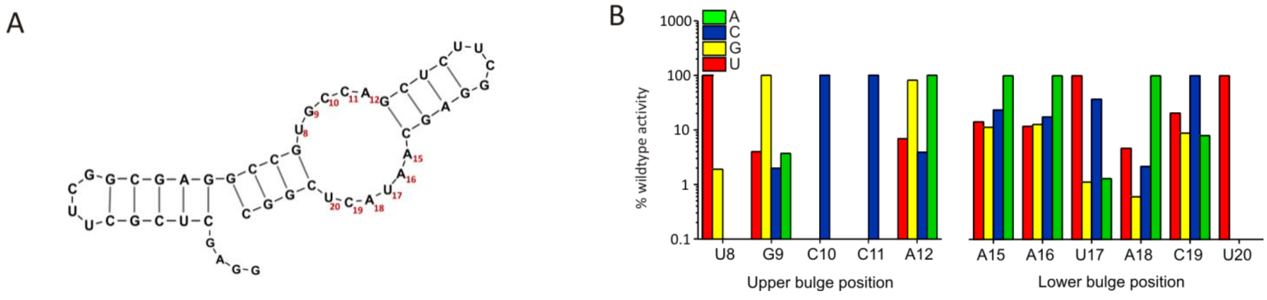

Supplementary Figure S9. Biochemical analysis data of 49mer DAse. (**A**) Numbering of nucleotides in the catalytic pocket. (**B**) Mutational data of DAse activity (adapted and modified from (21), involving information from (22). Numbering is according to the convention of (6). The wildtype DAse was mutated at all positions of upper and lower bulge, one at a time, to all possible alternative nucleotides. Reactivity was measured and normalized to wildtype activity.

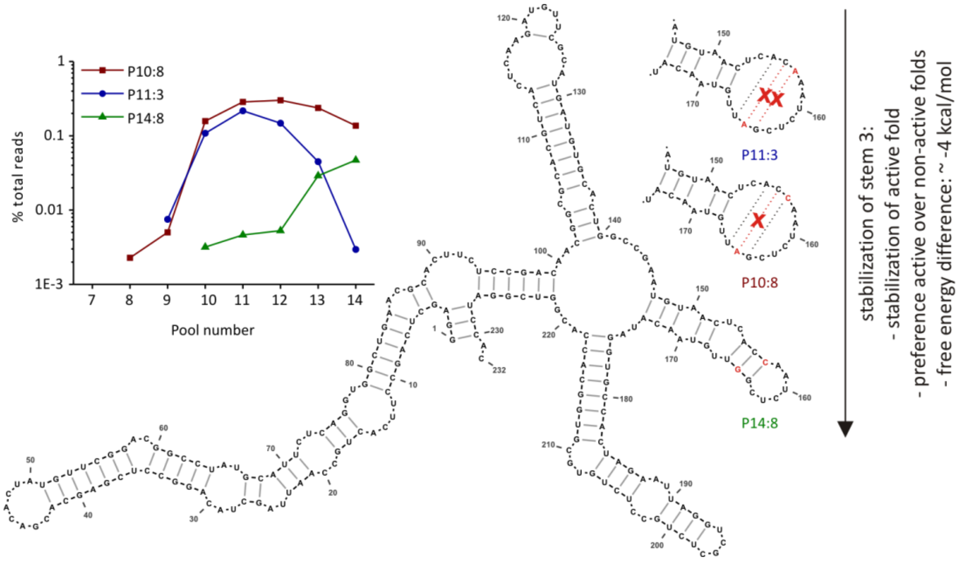

Supplementary Figure S10. Example of MIRzyme evolution by mutation. The substitutions of single nucleotides situated in one of the stem-loop structures of the active fold stabilize this fold and prevent the formation of alternative, inactive secondary structures.

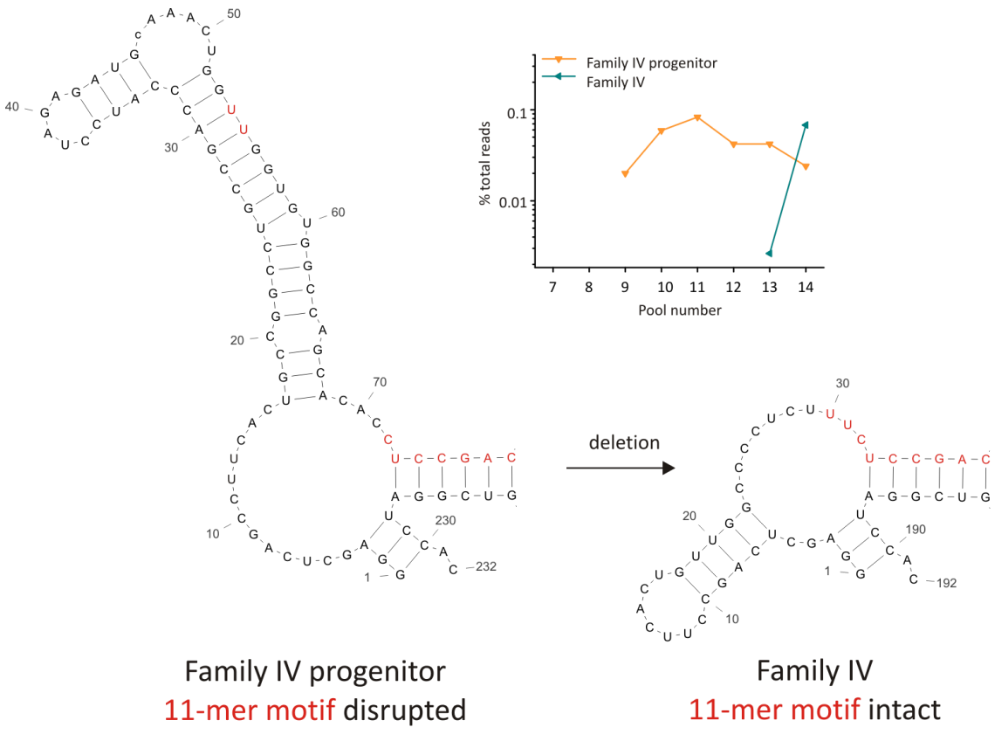

Supplementary Figure S11. Example of MIRzyme evolution by deletion. The deletion of large parts of the stem and loop structure between nt 17 and nt 68 of the family IV progenitor sequence (abundance shown in orange in the graph), results in a slightly different predicted secondary structure for the members of family IV (abundance shown in green), with the reconstitution of an intact 11-mer motif 5’-UUCUCCGACAA-3’ (nucleotides belonging to this motif shown in red), which has been associated with high catalytic activity previously (3). This explains the greater fitness of family IV members in contrast to members of the progenitor family.

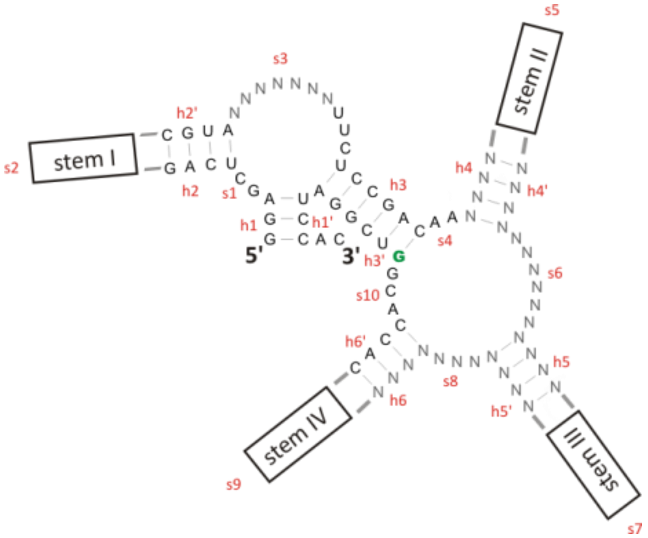

Supplementary Figure S12. MIRzyme secondary structure with denotation as in the RNABOB descriptors. This design is based on the family information from the original selection (3). The extensions of stems I, II, III and IV were found to differ in length as well as in sequence information and were therefore defined as single-stranded regions s2, s5, s7 and s9, respectively, in the RNABOB descriptor.

Supplementary Tables

Supplementary Table S1. Barcoding primers. (All primers were custom synthesized by Biomers).

Selection & pool number	Forward primer sequence	Reverse primer sequence	Barcode
**DAse 1**	GCAAGGTCTAATACGACTCACTATAGGAGC	GCAAGGGTGGATCCGACCGTGGTGCC	GCAAGG
**DAse 2**	TGCCATTCTAATACGACTCACTATAGGAGC	TGCCATGTGGATCCGACCGTGGTGCC	TGCCAT
**DAse 3**	AAGCGATCTAATACGACTCACTATAGGAGC	AAGCGAGTGGATCCGACCGTGGTGCC	AAGCGA
**DAse 4**	CCTTAGTCTAATACGACTCACTATAGGAGC	CCTTAGGTGGATCCGACCGTGGTGCC	CCTTAG
**DAse 5**	GGCCTGTCTAATACGACTCACTATAGGAGC	GGCCTGGTGGATCCGACCGTGGTGCC	GGCCTG
**DAse 6**	TTCTCCTCTAATACGACTCACTATAGGAGC	TTCTCCGTGGATCCGACCGTGGTGCC	TTCTCC
**DAse 7**	CTAGCTTCTAATACGACTCACTATAGGAGC	CTAGCTGTGGATCCGACCGTGGTGCC	CTAGCT
**DAse 8**	TATAATTCTAATACGACTCACTATAGGAGC	TATAATGTGGATCCGACCGTGGTGCC	TATAAT
**DAse 9**	ATACGGTCTAATACGACTCACTATAGGAGC	ATACGGGTGGATCCGACCGTGGTGCC	ATACGG
**DAse 10**	CTTGTATCTAATACGACTCACTATAGGAGC	CTTGTAGTGGATCCGACCGTGGTGCC	CTTGTA
**MIRzyme 1**	CACTCATCTAATACGACTCACTATAGGAGC	CACTCAGTGGATCCGACCGTGGTGCC	CACTCA
**MIRzyme 2**	AAGACTTCTAATACGACTCACTATAGGAGC	AAGACTGTGGATCCGACCGTGGTGCC	AAGACT
**MIRzyme 3**	CCGCAATCTAATACGACTCACTATAGGAGC	CCGCAAGTGGATCCGACCGTGGTGCC	CCGCAA
**MIRzyme 4**	GGCACATCTAATACGACTCACTATAGGAGC	GGCACAGTGGATCCGACCGTGGTGCC	GGCACA
**MIRzyme 5**	TTCGAATCTAATACGACTCACTATAGGAGC	TTCGAAGTGGATCCGACCGTGGTGCC	TTCGAA
**MIRzyme 6**	ACTCTCTCTAATACGACTCACTATAGGAGC	ACTCTCGTGGATCCGACCGTGGTGCC	ACTCTC
**MIRzyme 7**	CGTACGTCTAATACGACTCACTATAGGAGC	CGTACGGTGGATCCGACCGTGGTGCC	CGTACG
**MIRzyme 8**	GTAGAGTCTAATACGACTCACTATAGGAGC	GTAGAGGTGGATCCGACCGTGGTGCC	GTAGAG
**MIRzyme 9**	TACAGCTCTAATACGACTCACTATAGGAGC	TACAGCGTGGATCCGACCGTGGTGCC	TACAGC
**MIRzyme 10**	AGTTCCTCTAATACGACTCACTATAGGAGC	AGTTCCGTGGATCCGACCGTGGTGCC	AGTTCC
**MIRzyme 11**	CTCAGATCTAATACGACTCACTATAGGAGC	CTCAGAGTGGATCCGACCGTGGTGCC	CTCAGA
**MIRzyme 12**	GAGTGGTCTAATACGACTCACTATAGGAGC	GAGTGGGTGGATCCGACCGTGGTGCC	GAGTGG
**MIRzyme 13**	TCCCGATCTAATACGACTCACTATAGGAGC	TCCCGAGTGGATCCGACCGTGGTGCC	TCCCGA
**MIRzyme 14**	ATGAGCTCTAATACGACTCACTATAGGAGC	ATGAGCGTGGATCCGACCGTGGTGCC	ATGAGC

Supplementary Table S2. Primers for site-directed mutagenesis. Mutated nucleotides are highlighted in red and underlined. (All primers were custom synthesized by Biomers).

Primer	Sequence	Sense	Sequence element	Position	Nucleotide mutated to
**mutprimer_s6_GtoC**	GAGTATAGCCCAACGCCGTG	fwd	s6	4	C
**mutprimer_s6_GtoU**	GAGTATAGCCTAACGCCGTG	fwd	s6	4	U
**mutprimer_s6_GtoA**	GAGTATAGCCAAACGCCGTG	fwd	s6	4	A
**mutprimer_s6_AtoC**	GAGTATAGCCGCACGCCGTG	fwd	s6	5	C
**mutprimer_s6_AtoG**	GAGTATAGCCGGACGCCGTG	fwd	s6	5	G
**mutprimer_s6_AtoU**	GAGTATAGCCGTACGCCGTG	fwd	s6	5	U
**primer_s6_rev**	GCTAAACTATATTGCTAGCGCAC	rev	s6	-	-
**mutprimer_s8_GtoA**	GTGGATCCGACCGTGGTGCCAGACAGGCACCTTACACC	rev	s8	2	A
**mutprimer_s8_GtoC**	GTGGATCCGACCGTGGTGCCAGACAGGCACCTGACACC	rev	s8	2	C
**mutprimer_s8_GtoU**	GTGGATCCGACCGTGGTGCCAGACAGGCACCTAACACC	rev	s8	2	U
**primer A**	TCTAATACGACTCACTATAGGAGCTCAGCCTTCACTGC	fwd			
**primer B**	GTGGATCCGACCGTGGTGCC	rev			

Supplementary Table S3. Pool diversity in the DAse selection by total reads. Measured by the percentage of total reads represented by the top 1 sequence, the 10 most abundant sequences, by sequences with > 2 reads, sequences with > 10 reads, and sequences covering > 0.01% of total reads.

Pool number	% total reads top 1	% total reads top 10	% total reads >2 reads	% total reads >10 reads	% total reads > 0.01%	Total reads
**1**	0%	0%	0%	0%	0%	43136
**2**	0%	0%	0%	0%	0%	80381
**3**	0%	0%	0%	0%	0%	75477
**4**	0%	0%	0%	0%	0%	93084
**5**	0%	0%	0%	0%	0%	59273
**6**	5%	14%	37%	32%	33%	71618
**7**	11%	27%	75%	64%	64%	108006
**8**	10%	34%	79%	69%	70%	93931
**9**	13%	39%	81%	72%	73%	92126
**10**	12%	36%	79%	70%	70%	116503

Supplementary Table S4. Pool diversity in the DAse selection by distinct sequences. Measured by the percentage of distinct sequences covered by sequences with > 2 or > 10 reads, and by the ratio of distinct sequences over total reads.

Pool number	% distinct sequences > 2 total reads	% distinct sequences > 10 total reads	Distinct sequences	Distinct sequences / total reads	1-(distinct sequences /total reads)
**1**	0%	0%	42907	99%	1%
**2**	0%	0%	80032	100%	0%
**3**	0%	0%	75130	100%	0%
**4**	0%	0%	92671	100%	0%
**5**	0%	0%	58970	99%	1%
**6**	3%	1%	44855	63%	37%
**7**	11%	2%	28303	26%	74%
**8**	13%	3%	20565	22%	78%
**9**	13%	4%	18088	20%	80%
**10**	11%	3%	25689	22%	78%

Supplementary Table S5. Overview of different groups identified in the DAse selection pool 7 by multiple alignments. Groups of sequences were classified by the number of distinct members belonging to them. Within each class of groups, different numbers were calculated: the number of groups, the total number of distinct sequences covered by these groups, % of distinct sequences covered by these groups, number of total reads covered by these groups, % of total reads covered by these groups, the average number of reads per distinct sequence within these groups, the average percentage of reads covered by the dominating sequence of each group, and the number of typical DAse groups.

Number of members	>1000	101-1000	21-100	11-20	3-10	2	Non-aligned	Total
**Number of groups**	2	46	102	75	330	204	5690	759
**Distinct sequences covered**	3426	11062	4912	1121	1677	408	5690	28296
**% distinct sequences**	12%	39%	17%	4%	6%	1%	20%	100%
**Total reads covered by groups**	26561	54320	14821	2644	3195	583	5876	108000
**% total reads**	25%	50%	14%	2%	3%	1%	5%	100%
**Average reads / sequence**	7.8	4.9	3.0	2.4	1.9	1.4	1.0	-
**Average % reads of dominating sequence**	57%	58%	57%	55%	55%	64%	0%	-
**Number of typical DAse groups**	2	35	52	45	166	90	433	823

Supplementary Table S6. Overview of different groups identified in the DAse selection pool 10 by multiple alignments. Groups of sequences were classified by the number of distinct members belonging to them. Within each class of groups, different numbers were calculated: the number of groups, the total number of distinct sequences covered by these groups, % of distinct sequences covered by these groups, number of total reads covered by these groups, % of total reads covered by these groups, the average number of reads per distinct sequence within these groups, the average percentage of reads covered by the dominating sequence of each group, and the number of typical DAse groups.

Number of members	>1000	101-1000	21-100	11-20	3-10	2	Non-aligned	total
**Number of groups**	5	28	37	24	83	57	3430	234
**Distinct sequences covered**	10164	9569	1638	356	418	114	3430	25689
**% distinct sequences**	40%	37%	6%	1%	2%	0.4%	13%	100%
**Total reads covered by groups**	64665	42897	4010	636	719	114	3462	116503
**% total reads**	56%	37%	3%	1%	1%	0.1%	3%	100%
**Average reads / sequence**	6.4	4.5	2.4	1.8	1.7	2.5	1.0	-
**Average % reads of dominating sequence**	39%	44%	39%	39%	41%	55%	0%	-
**Number of typical DAse groups**	5	25	26	20	56	28	152	312

Supplementary Table S7. Average percentage of each error type among 23 groups of DAse ribozymes.

	to A	to C	to G	to T
**from A**	-	2%	19%	4%
**from C**	2%	-	1%	19%
**from G**	8%	2%	-	3%
**from T**	2%	14%	2%	-
**Insertions**		8%		
**Deletions**		14%		

Supplementary Table S8. Average percentage of the two different types of transitions and transversions, as well as of indel-type errors. Transitions were classified as purine-purine (R-R) or pyrimidine-pyrimidine (Y-Y) transitions, while transversions were classified as purine-pyrimidine (R-Y) or pyrimidine-purine (Y-R) transversions. Discrepancies with the addition of values from Supplementary Table S7 result from rounding.

Error type	Percentage
**R-R**	27%
**Y-Y**	34%
**R-Y**	11%
**Y-R**	6%
**indel**	23%

Supplementary Table S9. Pool diversity in the MIRzyme selection by total reads. Measured by the percentage of total reads represented by the top 1 sequence, the 10 most abundant sequences, by sequences with > 2 reads, sequences with > 10 reads, and sequences covering > 0.01% of total reads.

Pool number	% total reads top 1	% total reads top 10	% total reads >2 reads	% total reads >10 reads	% total reads > 0.01%	Total reads
**1**	0.00%	0.04%	0.00%	0.00%	0.00%	52572
**2**	0.00%	0.03%	0.00%	0.00%	0.00%	58108
**3**	0.01%	0.04%	0.01%	0.00%	0.00%	57897
**4**	0.01%	0.11%	0.00%	0.00%	0.00%	18429
**5**	0.02%	0.16%	0.00%	0.00%	1.4%	12698
**6**	0.00%	0.02%	0.00%	0.00%	0.00%	99225
**7**	0.02%	0.05%	0.02%	0.00%	0.02%	57678
**8**	0.13%	0.52%	0.61%	0.39%	0.51%	32304
**9**	0.81%	3.4%	6.8%	4.2%	6.8%	29124
**10**	0.46%	2.1%	6.8%	4.1%	5.5%	45503
**11**	0.32%	1.5%	6.1%	3.7%	4.3%	65674
**12**	0.26%	1.3%	2.8%	1.4%	4.9%	14202
**13**	0.29%	1.3%	3.5%	1.5%	3.5%	28240
**14**	0.33%	1.3%	4.9%	1.9%	2.8%	52077

Supplementary Table S10. Pool diversity in the MIRzyme selection by distinct sequences. Measured by the percentage of distinct sequences covered by sequences with > 2 or > 10 reads, and by the ratio of distinct sequences over total reads.

Pool number	% distinct sequences > 2 total reads	% distinct sequences > 10 total reads	Distinct sequences	Distinct sequences / total reads	1-(distinct sequences /total reads)
**1**	0.00%	0.00%	52371	99.6%	0.4%
**2**	0.00%	0.00%	57852	99.6%	0.4%
**3**	0.00%	0.00%	57696	99.7%	0.3%
**4**	0.00%	0.00%	18374	99.7%	0.3%
**5**	0.00%	0.00%	12653	99.6%	0.4%
**6**	0.00%	0.00%	98895	99.7%	0.3%
**7**	0.00%	0.00%	57489	99.7%	0.3%
**8**	0.06%	0.01%	32017	99.1%	0.9%
**9**	0.73%	0.09%	27029	92.8%	7.2%
**10**	0.79%	0.13%	42172	92.7%	7.3%
**11**	0.72%	0.12%	61275	93.3%	6.7%
**12**	0.41%	0.08%	13726	96.6%	3.4%
**13**	0.54%	0.06%	27089	95.9%	4.1%
**14**	0.80%	0.06%	49157	94.4%	5.6%
